# On building a diabetes centric knowledge base via mining the web

**DOI:** 10.1186/s12911-019-0771-6

**Published:** 2019-04-09

**Authors:** Fan Gong, Yilei Chen, Haofen Wang, Hao Lu

**Affiliations:** 1 Shanghai Shuguang Hospital Affiliated to Shanghai University of Traditional Chinese Medicine, Pu’an Road, Shanghai, China; 2 Shanghai Leyan Technologies Co. Ltd, No. 1028 Panyu Road, Shanghai, China

**Keywords:** Knowledge extraction, Knowledge fusion, Diabetes-centric KB

## Abstract

**Background:**

Diabetes has become one of the hot topics in life science researches. To support the analytical procedures, researchers and analysts expend a mass of labor cost to collect experimental data, which is also error-prone. To reduce the cost and to ensure the data quality, there is a growing trend of extracting clinical events in form of knowledge from electronic medical records (EMRs). To do so, we first need a high-coverage knowledge base (KB) of a specific disease to support the above extraction tasks called *KB-based Extraction*.

**Methods:**

We propose an approach to build a diabetes-centric knowledge base (a.k.a. DKB) via mining the Web. In particular, we first extract knowledge from semi-structured contents of vertical portals, fuse individual knowledge from each site, and further map them to a unified KB. The target DKB is then extracted from the overall KB based on a distance-based Expectation-Maximization (EM) algorithm.

**Results:**

During the experiments, we selected eight popular vertical portals in China as data sources to construct DKB. There are 7703 instances and 96,041 edges in the final diabetes KB covering diseases, symptoms, western medicines, traditional Chinese medicines, examinations, departments, and body structures. The accuracy of DKB is 95.91%. Besides the quality assessment of extracted knowledge from vertical portals, we also carried out detailed experiments for evaluating the knowledge fusion performance as well as the convergence of the distance-based EM algorithm with positive results.

**Conclusions:**

In this paper, we introduced an approach to constructing DKB. A knowledge extraction and fusion pipeline was first used to extract semi-structured data from vertical portals and individual KBs were further fused into a unified knowledge base. After that, we develop a distance based Expectation Maximization algorithm to extract a subset from the overall knowledge base forming the target DKB. Experiments showed that the data in DKB are rich and of high-quality.

## Background

### Introduction

Diabetes is one of the major threats to human health, which is expected to affect 552 million people by 2030. Especially, the rate of prevalence of diabetes is high in Asian countries. About 92.3 million and 63 million people in China and India suffer from this metabolic disorder respectively.

In this context, diabetes has become a hot topic in life science researches recently and there is a growing trend of using EMRs as a source of clinical information to support the analytical procedures. It has been advocated that EMR adoption is a key to solving problems related to quality of care, clinical decision support, and reliable information flow among individuals and departments participating in patient care. Randall et al. [1] compared achievements of and improvements in quality standards for diabetes using EMRs with those using paper records at practices. Suna et al. [2] designed and constructed a diabetes knowledge graph from EMRs and proposed a deep neural network for its completion. Their findings supported the meaningful use of EMRs to improve the quality of care.

To realize the potential of EMRs, we need to extract knowledge in form of structured information from the huge amount of unstructured textual content in EMRs. Such extraction task is challenging and usually requires a lot of manual efforts. For example, most EMRs record a narrative describing the history of present illness. Clinical researches retrieve this information by employing domain experts to manually curate such narratives. This process can be both error-prone and labor-intensive. To reduce the cost and to ensure the data quality, a high-coverage KB of a specific disease is needed to support the above KB-based extraction task.

A variety of works aimed at using analysis and mining techniques to provide high quality, well-informed and cost-effective knowledge. Yang et al. [3] built T2D@ZJU, which was retrieved from pathway databases, protein-protein interaction databases and literature. Krishnasamy [4] built DAPD, which was developed to link diabetes with genes, pathways and proteins. Rotmensch Maya et al. [5] explored an automated process to learn high quality knowledge bases linking diseases and symptoms directly from EMRs. However, these works failed to capture non-standard terminologies or abbreviations in their resulting knowledge bases.

On the other hand, there are quite a few vertical portals provide information related to healthcare on the Web. They focus on providing a relatively narrow range of medical services and share information about various medical knowledge. We consider to make use of all these sources together and fuse them into a unified KB, which can have a large coverage of different aspects of diabetes knowledge. Such kind of diabetes-centric resource can also be further extended and implemented on real-world applications to benefit the public health more.

In this paper, we propose an approach to constructing a DKB via mining the Web. We introduce an integrated approach in order to tackle the challenges during the KB construction. More specifically, the contributions are stated as follows:

- The data on the Web are usually full of noise, which means they may be redundant, complementary, and sometimes have conflicts in some values. To ensure the high-quality of the data in our built KB, we describe a detailed method for *knowledge extraction* from vertical portals. Such portals provide manually editing information in the form of semi-structured data. They are transformed into structured data via a *wrapper-based extraction strategy* and stored in a relational database following the *vertical partitioning design*.
- Data sets used for knowledge construction are scattered in various prominent sources which calls for fusing distributed knowledge in a unified representation way. We adopt an *ontological structure* as the knowledge representation of our KB, which includes a finer-grained schema and actual data following it. We map all the relational tables to the predefined KB structure via a *D2R mapping strategy*. In order to integrate the scattered extracted data, we propose a knowledge fusion method. Concretely, we first cleanse the extracted data, especially resolving the conflicting class description of the same instance. After that, we follow a state-of-the-art mapping approach to detecting equivalent instance matchings.
- We develop a distance-based EM algorithm to extract a subset (all related to diabetes) from the unified KB forming the target DKB. It consists of an initial step, an expectation step and a maximization step to extract highly relevant knowledge under the topic “diabetes”. Experiments also prove that the DKB we built is of good quality and robust to be further extended with more new knowledge.

The rest of the paper is organized as follows. The problem definition and knowledge base schema are introduced in “Problem Definition” section. “Related Work” section lists the related work. “Methods” section presents the knowledge base construction module. “Results” section shows the experiment results. In “Discussions” section, we provide the analysis of the method and the experiments. Conclusions and future work are discussed in “Conclusions” section.

### Problem Definition

We aim at building a unified KB targeting diabetes. In this part, we start with a brief introduction of the definition of our proposed KB, then give an overview of the data sources used for its construction and finally provide the overall workflow of our approach to build the KB.

#### Brief Overview of KB

**Fig. 1:**
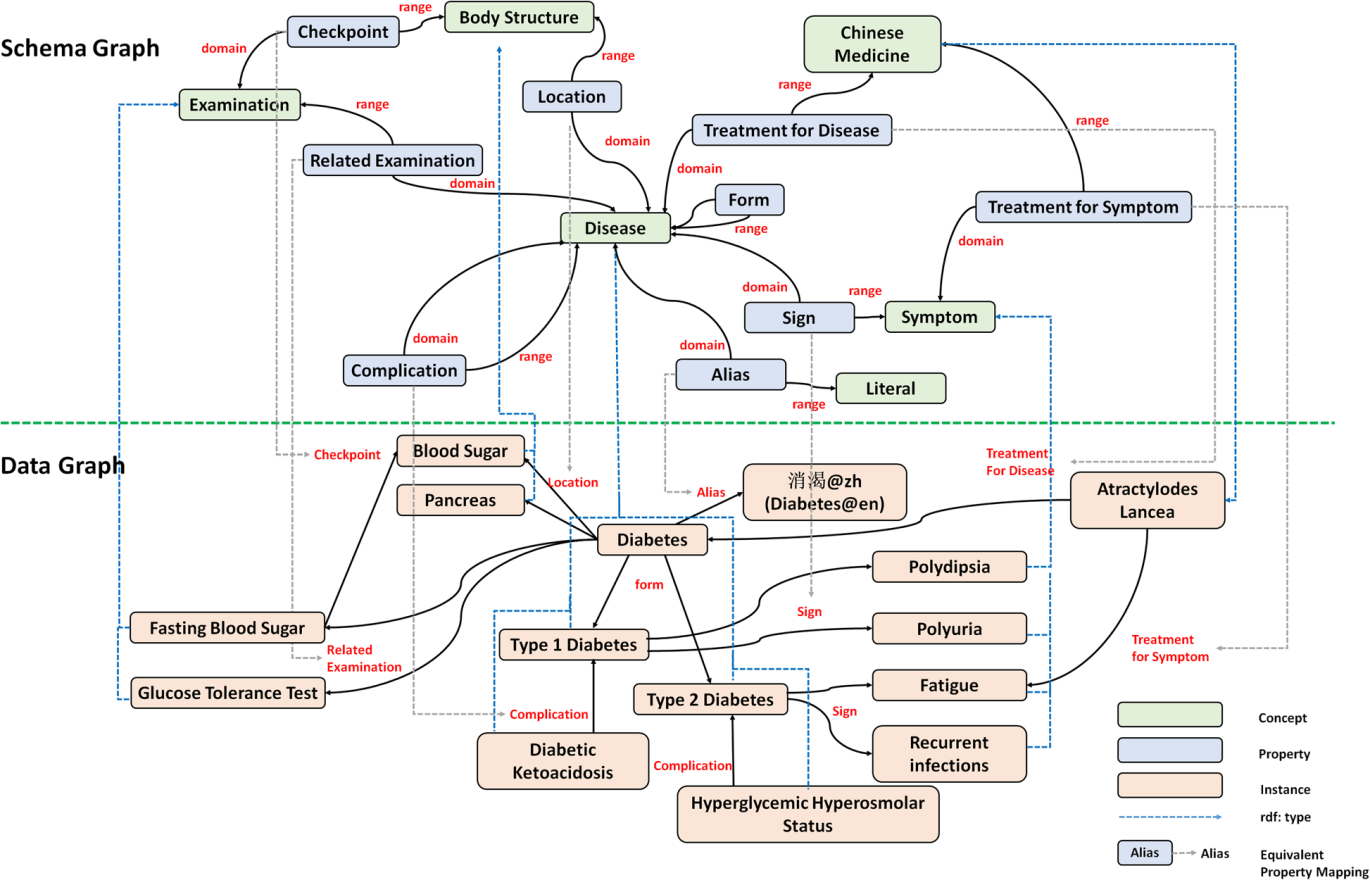
A complete schema graph and a subset of the corresponding data graph

We use an ontological structure *O* to represent the target DKB, which is shown in Fig. 1 The KB is denoted as *O*=<*G*_*s*_,*G*_*d*_> where *G*_*s*_ is a schema graph and *G*_*d*_ is a data graph. We distinguish a schema graph *G*_*s*_ capturing the schema-level knowledge of the DKB from *G*_*d*_ representing the actual data which follows the schema designed in *G*_*s*_. Here, we adapt three popular knowledge representation languages recommended by W3C (i.e. RDF [6], RDFS [7], and OWL [8]) as the basis of our KB definition.

##### **Definition 1**

The data graph *G*_*d*_ is represented as a tuple (*V,L*,*E*) where

- *V* is a finite set of vertices. *V* is conceived as the disjoint union $V_{I} \biguplus V_{V}$ with *I*-vertices *V*_*I*_ (representing instances), and *V*-vertices *V*_*V*_ (data values).
- *L* is a finite set of edge labels, subdivided by $L = L_{R} \biguplus L_{A} \biguplus \{{rdfs:type}\}$, where *L*_*R*_ represent inter-entity edges and *L*_*A*_ stand for entity-attribute assignments.
- *E* is a finite set of edges of the form *e*(*v*_1_,*v*_2_) with *v*_1_,*v*_2_∈*V* and *e*∈*L*. Moreover, the following restrictions are distinguished:
  - *e*∈*L*_*R*_ if and only if *v*_1_,*v*_2_∈*V*_*I*_,
  - *e*∈*L*_*A*_ if and only if *v*_1_∈*V*_*I*_ and *v*_2_∈*V*_*V*_, and
  - *e*=*rdf*:*type* denotes the membership of an instance in a particular class.

##### **Definition 2**

The schema graph *G*_*s*_ is represented as a tuple (*V,L*,*E*) where

- *V* is a finite set of vertices. Here, *V* is conceived as the disjoint union $V_{C} \biguplus V_{R} \biguplus V_{A} \biguplus V_{D}$ with *C*-vertices *V*_*C*_ (representing classes), *R*-vertices *V*_*R*_ (relations), *A*-vertices *V*_*A*_ (attributes), and *D*-vertices *V*_*D*_ (data types).
- *L* comprises of edge labels including {*rdfs*:*domain,rdfs*:*range,rdfs*:*subclassOf,owl*:*disjointWith*}.
- *E* is a finite set of edges of the form *e*(*v*_1_,*v*_2_) with *v*_1_,*v*_2_∈*V* and *e*∈*L*. Moreover, the following restrictions apply:
  - *e*=*rdfs*:*domian* if and only if $v_{1} \in V_{A} \bigcup V_{R}$, and *v*_2_∈*V*_*C*_,
  - *e*=*rdfs*:*range* if and only if *v*_1_∈*V*_*A*_,*v*_2_∈*V*_*D*_ or *v*_1_∈*V*_*R*_,*v*_2_∈*V*_*C*_,
  - *e*=*rdfs*:*subclassOf* if and only if *v*_1_,*v*_2_∈*V*_*C*_, and
  - *e*=*owl*:*disjointWith* if and only if *v*_1_,*v*_2_∈*V*_*C*_ and *v*_1_∩*v*_2_=*∅*.

Figure 1 lists all the important classes as well as properties used to model the diabetes domain. We define seven classes namely “Disease”, “Symptom”, “Traditional Chinese Medicine (TCM)”, “Western Medicine”, “Department”, “Body Structure” and “Examination”. These classes are selected manually from the highly frequent phrases representing key concepts collected from vertical portals. In addition, we manually define ten different relations in a top-down manner. According to a recent report on the frequently-asked questions on diabetes in WebMD [9] and several academic diabetes self-management questionnaire [10], we find that the selected classes and relations can meet the need of capturing diabetes knowledge (including non-standard terminologies or abbreviations). Take the edge “Sign” as an example, it connects a disease “Type 1 Diabetes” with a symptom “Polydipsia”, which represents the symptom is a sign of the disease. Another example can be taken from the Figure is the property “Alias”, which is treated as an entity-attribute relation. We can infer from such relation that “” is a synonym of “ (diabetes)”.

Besides the named classes and typed properties, we also define several axioms. Five predefined types of edges, i.e. *rdfs:domian*, *rdfs:range*, *rdfs:subclassOf*, *owl:disjointWith* and *rdf:type* are included in our KB, in which each type has a particular interpretation. A *rdfs:domain* label describes a constraint that the subject of a given relation or an attribute *p* must belong to a given class *c*, which is represented as a triple <*p,rdfs*:*domain,c*>. Similarly a *rdfs:range* label declares the value of a given relation or an attribute must belong to a given class *c* or to a data value *d* in the specified data range, which is organized as a triple <*p,rdfs*:*range,c*> or <*p,rdfs*:*range,d*>. A *rdfs:subclassOf* label is used to define the class hierarchy and a *owl:disjointWith* label asserts that two classes have no instances in common. *rdf:type* serves as the connections between the schema graph *G*_*s*_ and the data graph *G*_*d*_, which link the instances to their corresponding concepts.

For example, “Sign” is defined as a relation, whose *rdfs:domian* is the class “Disease” and *rdfs:range* is the class “Symptom”. In addition, these two classes are declared as *owl:disjointWith* so that the instance “Diabetes” of the class “Disease” cannot be an instance of “Symptom”. Moreover, *rdfs:subclassOf* is used to indicate that the class “Medicine” has two subclass “Traditional Chinese Medicine” and “Western Medicine”. “Atractylodes Lancea” is declared as an instance of the class “Traditional Chinese Medicine” through *rdfs:type*.

#### Data Sources Used for DKB Construction

**Fig. 2:**
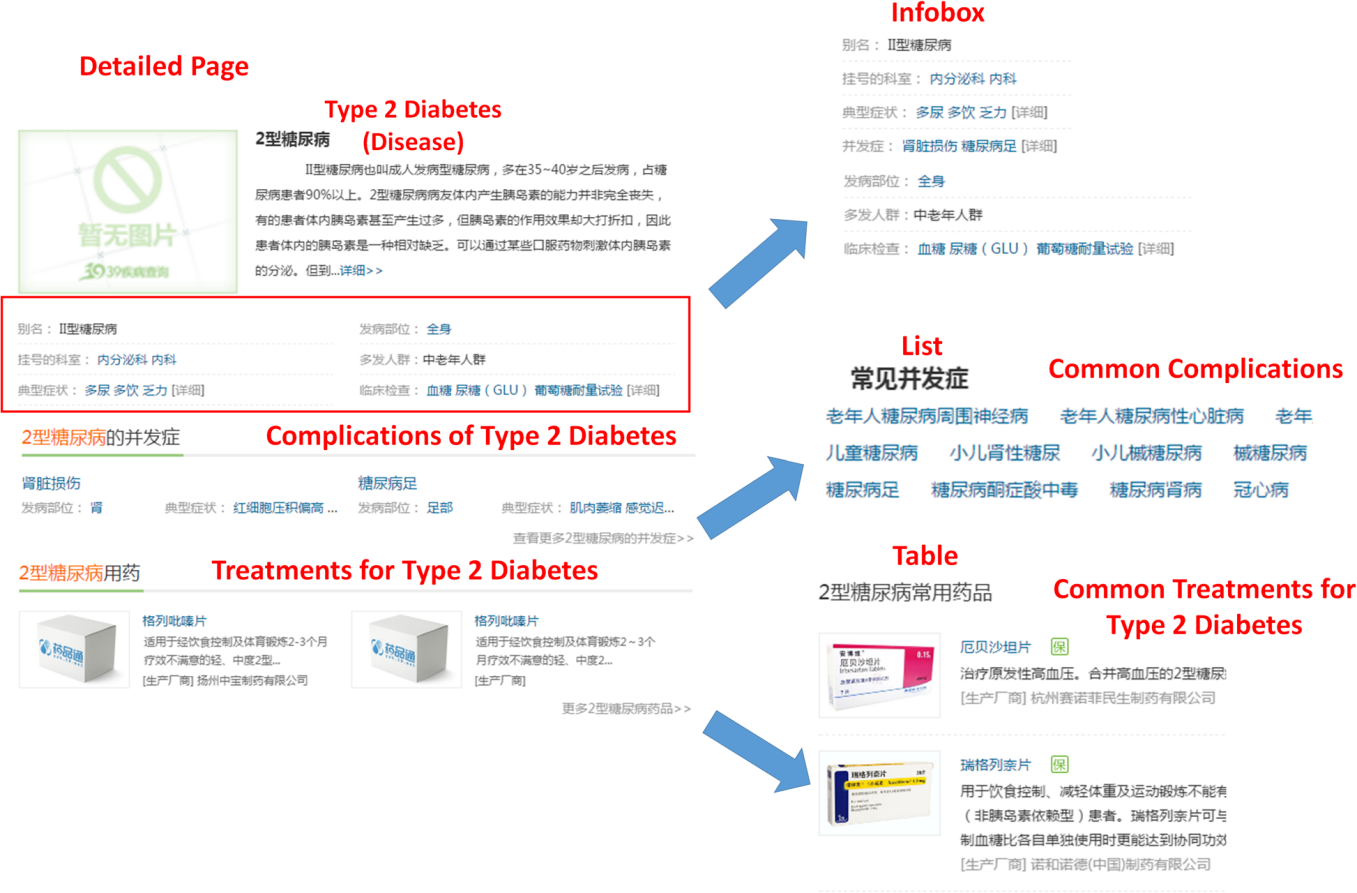
The organization of pages of a vertical portal used for knowledge extraction

Since different vertical portals share very similar structures, we just take the representative example “39 (39Health)” to explain the details of pages and their structures used for knowledge extraction. As shown in Fig. 2, data in vertical portals are usually semi-structured and content providers usually design several *navigation pages* to gather instances within the same class together. Such page can also be regarded as a Web *table* in which each row contains a structural summary of a particular instance as well as an external link to a detailed page describing the instance-related information. We list a navigation page for diseases in the Fig. 2. It provides a short summary of “Type 2 Diabetes” as well as an external link “http://jbk.39.net/iixtnb/” to a detailed page in which more knowledge corresponding to “Type 2 Diabetes” is displayed. Besides, in order to distinguish different instances in the same portal, content providers design a URL pattern “http://jbk.39.net/identifier” where “{identifier}” stands for the label of a particular instance, such as using “iixtnb” as the identifier of “Type 2 Diabetes”. Thus, each instance is disambiguated and we can get a richer information through looking inside its detailed page.

We find three possible entries for knowledge extraction in these detailed pages, namely *lists*, *tables* and *infoboxes*. *List* is a parallel structure whose precedent word is usually representative for its following description. As shown in the Figure, the complications of diabetes are organized in a list which has a topic “Common Complications” on the first line. It indicates that the following instances are describing the corresponding complications of diabetes. *Table* is usually organized similarly as list, which contains the topic sentence on the first row and its corresponding instances in the following rows. *Infobox* is an array of key-value pairs in which each key represents a property and the value corresponds to one or several property values (either data values or instances). Each instance (“Type 2 Diabetes” from the example) serves as the subject of a triple connecting with the property along with one of its distinct values. The potential relation described by each key-value pair can be inferred from its property label.

#### Workflow

**Fig. 3:**
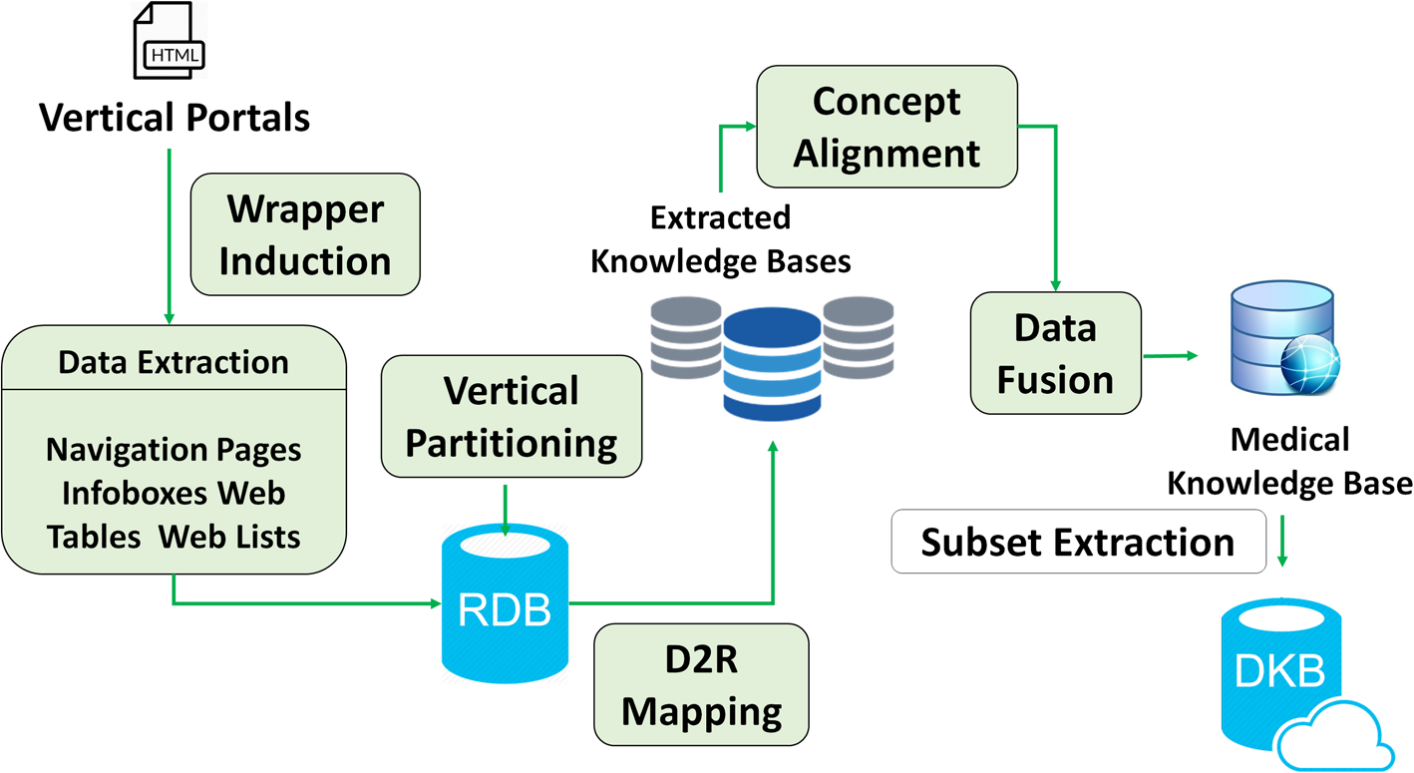
Overall workflow of our approach to constructing DKB

We now provide a workflow to depict the process of our approach, which is shown in Fig. 3. Since vertical portals have a similar data structure and can only be accessed through Web pages, we design different wrappers to extract information from *navigation pages*, *infoboxes*, *Web tables* and *Web lists*. The extracted data are then stored in a relational database according to the vertical partitioning design [11]. All triples are rewritten into several two-column tables. For each table (representing a specific property), the first column contains the subjects that define the property and the second column contains the values of that property for a given subject. After that, we apply a D2R (i.e. Database to RDF) step to map these tables in the database to the data graph *G*_*d*_ in the KB. For each portal, the extracted data are used to construct an independent KB so that we get several different KBs after the data extraction process. However, triples from different KBs may have complements, redundances or even conflicts between each other. Therefore, we provide a fusion strategy to integrate these separated KBs into a unified one. We propose a voting algorithm including self-conflicting detection in order to improve the quality of the extracted data. Then we adopt a standard process of instance matching [12] in order to identify the resources described in different datasets that correspond to the same real-world entity. Finally, we introduce a distance based EM (i.e. Expectation-Maximization) algorithm to extract a subset targeting the diabetes from the overall KB, which is so called *DKB*.

### Related Work

There are three lines of research related to the problem we solve. Details are discussed as follows respectively.

- **Existing Knowledge Base Constructed Via Mining the Web** Over the past decade, there emerged a number of automatically constructed large-scale knowledge bases via Web mining, which contain millions or even billions items of knowledge. Such knowledge bases usually employ information extraction techniques to extract knowledge from the Web (e.g. Wikipedia articles or general Web pages). Notable endeavors in the academic community include: Open Information Extraction [13], DBpedia [14], and YAGO [15]. In this phase, a number of large-scale Chinese knowledge bases have also emerged, including Zhishi.me [16], SSCO [17] and the commercial knowledge bases Sogou Zhilifang [18] and Baidu Zhixin [19] supporting Chinese search engines.
- **Medical Knowledge Base** There exist many different types of medical KBs. For example, UMLS [20] and SNOMED-CT [21] promote standardization and inter-operability for biomedical information systems and services. DrugBank [22] and SIDER [23] contain drug-related information. These knowledge bases are built and maintained manually with heavy human efforts. There are also some studies in the medical field which begins to construct a knowledge base by automatic algorithms. Knowlife [24] is a knowledge graph for the biomedical science which extracts and fuses data from scientific publications, encyclopedic health care portals and online communities. They used a distant supervision algorithm in the extraction phase and employed logical reasoning for consistency checking. Different from the above mentioned work, our extraction target focuses on diabetes and diabetes-related entities. Also, the types of data sources and methods used are quite different.
- **Diabetes Knowledge Base** T1Dbase [25], T2D-Db [26], T2DGADB [27] and T2D@ZJU [28] are active serving KBs for Type I and Type II diabetes. T1Dbase supports the Type I diabetes community with genetics and genomics of Type I diabetes susceptibility (T1D). The T2D-Db database provides an integrated platform for the better molecular level understanding of Type II diabetes mellitus and its pathologies. It manually created 330 candidate genes from the Pubmed literature and provided their corresponding information. T2DGADB collected 701 publications in T2D genetic association studies and T2D@ZJU contains heterogeneous connections associated with Type II diabetes. These databases concentrate on dealing with genetic association studies as well as more integrated resources involving gene expressions, pathways and protein-protein interactions. However, all the existing diabetes KBs are all counting on English resources and the presence of Chinese DKBs is rather limited, which is the focus of our work.

## Methods

In this section, we first introduce the method to extract semantic data in the form of triples from vertical portals. As a result, we get several individual KBs from these sites. In order to build a unified one, we describe a method to fuse these individual KBs with sophisticated fusion strategies. Finally, we develop a distance-based EM algorithm to extract DKB from the integrated KB.

### Knowledge Extraction

As the starting point of the extraction process, we manually select eight prevalent vertical portals on healthcare in China, namely “39 (39Health)” [29], “99 (99Health)” [30], “ (FhHealth)” [31], “ (Familydoctotr)” [32], “ (GlobalHospital)” [33], “ (PcBaby)” [34], “ (JianKe)” [35] and “ (120Ask)” [36]. All these sites have supplied HTML pages describing symptoms, diseases, departments, body structures and medicines so that we are provided with sufficient medical knowledge for constructing the DKB. The main difference among these sites is that only “ (JianKe)”, “ (120Ask)” and “ (39Health)” give detailed pages for describing examinations while we do not find such resources in the other five vertical portals. We will discuss the details of the class distribution among different portals in the experiment section.

#### Wrapper Induction

All these eight portals do not provide data dumps directly so that we have to extract relevant data through parsing the HTML pages. Therefore, we introduce wrapper induction, a sort of information extraction to extract knowledge from these semi-structured data on the Web pages. Dalvi et al. [37] presented a generic framework to learn wrappers across Web sites. Gentile et al. [38] presented a methodology called multi-strategy learning, which combines text mining with wrapper induction to extract knowledge from lists, tables, and Web pages.

Pages in these portals are usually automatically generated: data are stored in a back-end database management system (DBMS), and HTML pages are rendered using scripts from the content of the database. That is to say, the knowledge extraction process can be formulated as follows: “Given a set of sample HTML pages with a same structure belonging to a specific portal, which are regarded as seeds, we can generate a wrapper so as to find its corresponding structure. Then the wrapper is applied on more HTML pages to extract the source dataset from which the pages have been generated.” The extracted data is further mapped to the pre-designed tables in the relational database. The design of the table is carefully following the principle of *vertical partitioning*. Concretely speaking, we create a specific table for each edge *e*(*v*1,*v*2) defined in the data graph *G*_*d*_, where the triple <*v*1,*e,v*2> is rewritten into two columns <*v*1,*v*2> as a value pair. Since the URI of each resource is unique, we can leverage it as the identifier for each *v*∈*V*.

**Fig. 4:**
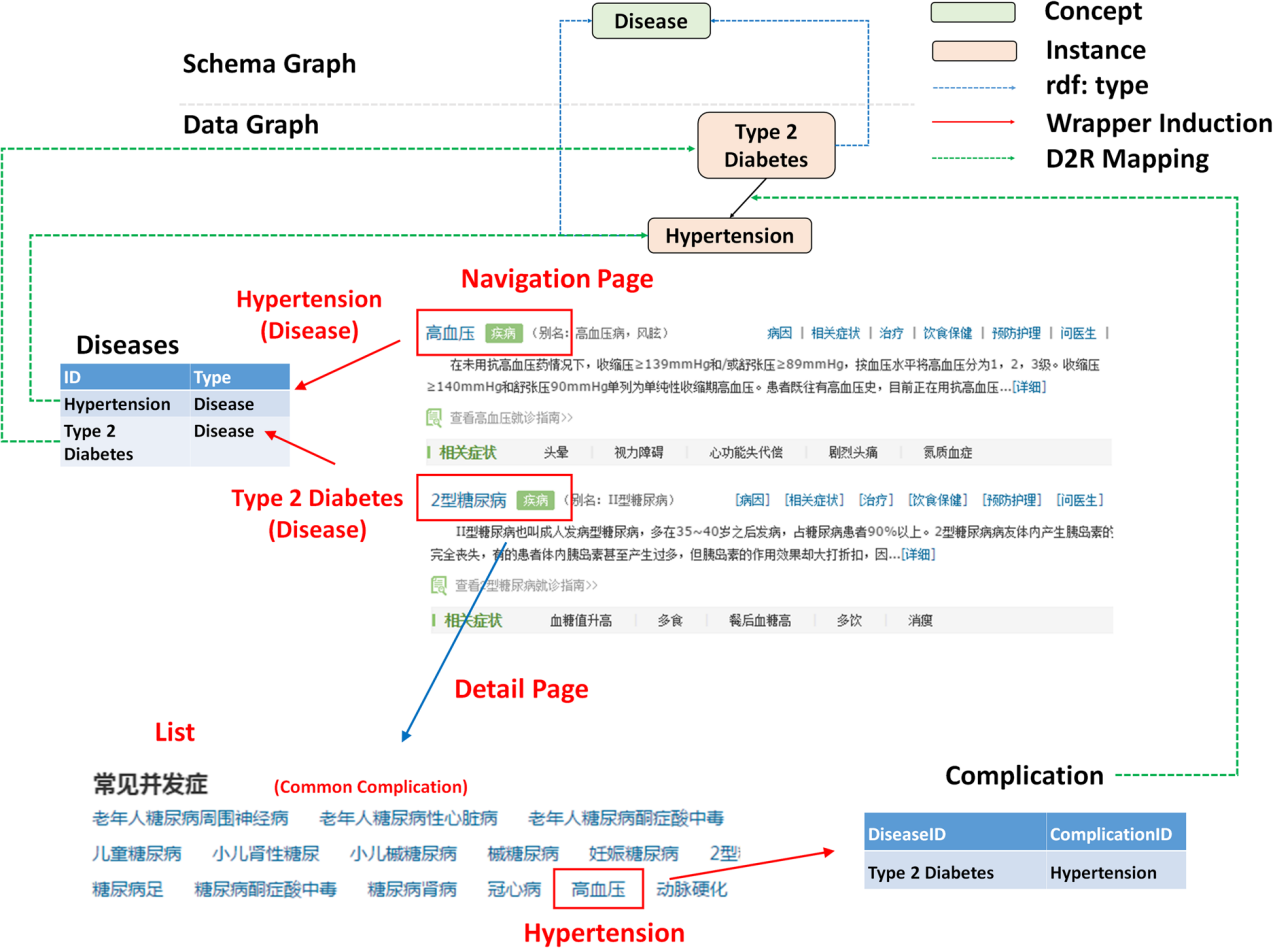
Example for Wrapper Induction and D2R Mapping

Note that the wrapper induction can only extract the structure of interest from a given page. The structure is comprised of a nested schema and corresponding values of fields defined in the schema. Any field might not be the same as the corresponding field name in the original database. Take the “Hypertension” in Fig. 2 as an example, it can be easily extracted as a member of the “common complications” through a wrapper. We need further select the corresponding table “Complication” in the relational database for it, which requires another strategy. Here, we propose a post-processing to tackle this problem. First, we collect a set of heuristic words *S*_*r*_={*w*_1_,*w*_2_,…,*w*_*n*_} for each relation *r* defined in the data graph *G*_*d*_, such as the word “ (complication)” for the relation “complication”. Next, we extract the topic sentence of all of the three entries, (i.e. the first line of the list, the first row of the table and the property label of the infobox). After that, we check the overlap between the extracted sentence with the pre-defined heuristic words set *S*_*r*_. If the sentence hits one of the heuristic words *w*_*n*_ in the set *S*_*r*_, the following extracted triples from that entry will be mapped to the table representing the relation *r*. Going back to the example in Fig. 4, “Hypertension” is a complication of “Type 2 diabetes” described in a *list* whose first line is “Common Complications”. Therefore, the following instances in the list are further rewritten into a table describing the property “Complication”. The table includes two columns where the first column is the identifier of “Type 2 diabetes” while “Hypertension” corresponds to its value as a second column.

#### D2R Mapping

After the wrapper induction step, we now mainly focus on transforming the relational data to our KB in form of triples, which is called database to RDF (a.k.a D2R) mapping. We use an existing D2R tool (i.e. D2RQ [39]) to deal with this problem. While the tool in general is to provide a virtual RDF layer on top of the relational database (RDB) [40], it can also export RDF triples from RDB. That is to say, the D2RQ Engine [41] does not convert the relational database into real RDF data, but uses a D2RQ Mapping file to map the database into virtual RDF formats.

In our database, each table stores exactly one property, and rows in the table contain subjects and the corresponding objects that use that property. We subdivide these tables into two groups: class tables and relation tables. For the former ones, they determine the class of each instance while the latter ones bridge the relation between instances.

First, a class table (e.g. the disease table) is structured from the navigation pages, which stores the property *rdf:type*. It has a primary key “ID” and an additional attribute “Type”. In order to declare the relation between the relation database schema and our KB, we manually create a D2R mapping file. Since a group of instances of “disease” all come from the same table, we can use one ClassMap and create the *rdf:type* statements with an object property bridge. Therefore, the first column is mapped to the subject and values in the second column as the corresponding property values. Here, the class is obtained by prefixing the values of the “Type” column with an ontology namespace.

For a relation table (e.g. the symptom table), we design a similar mapping file. More concretely, a d2rq:PropertyBridge is used to relate a database column “SymptomID” with a RDF property “symptom”. Thus, relations between instances of a disease (e.g. “diabetes”) and instances of a symptom (e.g. “polydipsia”) are created and mapped to the data graph.

### Knowledge Fusion

In “Brief Overview of KB” section, we have defined a mediated schema that provides a shared vocabulary for all instances in our KB. Therefore, in order to fuse these independent KBs into a unified one, we only need to focus on the data-level integration. To interrelate the elements of individual KBs, our graph model is extended with mappings.

- **Definition 1** A mapping *M* is a set of mapping assertions representing approximate correspondences between graph elements. Specifically, mapping assertions in *M* are of the form *m*(*v*1,*v*2) where *v*1,*v*2∈*V* are graph vertices from different datasets.
- **Definition 2** An integrated data graph *G*_*ID*_ is a tuple (*G*_*D*_,*M*_*D*_), where *G*_*D*_ is a finite set of data graphs and *M*_*D*_ is a set of approximate correspondences between data graph *I*-vertices. Each *m*∈*M*_*D*_ is called an individual mapping.

Web data is usually regarded as being full of noise. Many errors in the extracted candidate triples call for a strategy that can automatically decide the correctness of each triple, that is, whether the triple is consistent with the real world. Therefore, we first propose a pre-processing step to cleanse the data graph before obtaining the mappings between instances. In most cases, content providers of vertical portals edit a certain term describing a particular instance using their personal knowledge, which might lead to heterogeneous descriptions for the same instance. Take the instance “ (Hydronephrosis)” as an example, it is described as a symptom in “ (PcBaby)” while a disease in “39 (39Health)”. In our case, the class “disease” and “symptom” in the schema graph *G*_*s*_ are declared being disjointed with each other. That is to say, a specific instance should not belong to two disjoint classes at a time.

Statistically, over 90% of the conflicts come from the inconsistencies between the class “disease” and “symptom”, which are also hard for human beings to determine a clear boundary between them. Therefore, in this paper, we propose a probabilistic scoring algorithm to select the most likely class for each instance.

First of all, the gold standard ICD10 [42] is used to help separate the disease instances. It is a medical classification list of diseases provided by the WHO (World Health Organization). We collect a list in which each code is mapped to its related Chinese descriptions and aliases [43]. If the label of a certain instance hits a Chinese term in the list, its class will be determined as “disease”.

On the other hand, we adopt a voting strategy to help resolve the remaining inconsistencies. The main idea is, for a given instance, we tend to trust the class which has the most supported sources. Concretely, for a set of instances *v*_*I*1_,*v*_*I*2_,…,*v*_*In*_ sharing the same label, we collect their corresponding classes *v*_*c*1_,*v*_*c*2_,…,*v*_*cn*_ inferred from the triples <*v*_*In*_,*rdf*:*type,v*_*cn*_>. Then the voting strategy is formalized as:

1 $$  v^{*}_{c} = \underset{v_{c}}{\text{argmax}}{\frac{{\sum\nolimits}_{i=1}^{n} \mathbbm{1}(v_{c},v_{{ci}})}{n}}  $$

Where *𝟙* is the indicator which checks the equivalence between *v*_*c*_ and *v*_*ci*_. The class *v*_*c*_ with the highest score is selected as the result of the set of instances.

Since we have cleansed the class of each instance, the rest of the fusion work relies on instance matching. Thus, we follow a standard process established in the state-of-the-art mapping approaches in order to obtain high quality mappings *m*(*v*1,*v*2). The process can be decomposed into (1) engineering of similarity features, (2) selection of candidates, (3) computation of similarities (4) aggregation of similarities, and (5) derivation of correspondences based on the aggregated similarity values. For the similarity measures, we rely on existing, well-known measures that have been proven effective in state-of-the-art matching systems [44]. We primarily use simple, but effective measures based on syntactic and structural features. Here, correspondences between instances of the involved classes are computed. That is, only instances of the same class are processed at a time. If two instances do not share a same class, they are unlikely to be aligned.

### Extracting DKB from the Overall KB

The target DKB, denoted by *G*_*k*_, is regarded as a subset related with diabetes extracted from the overall KB. However, the real distribution of *G*_*k*_ in the whole data graph *G*_*d*_ is hard to be estimated. In order to separate *G*_*k*_ from *G*_*d*_, we develop a distance-based EM algorithm. The EM algorithm [45] usually consists of two steps, the Expectation step and the Maximization step. In our context, the key idea of the proposed algorithm is first to find the proper central vertices *v*_*c*_ in *G*_*k*_. Then we can select the vertices which are close enough to *v*_*c*_ and union them into *G*_*k*_. The whole algorithm is shown in Algorithm 1. Before introducing the Expectation-Maximization step, we first discuss the initialization strategy of our algorithm.

- **Initial step** We manually collect a bunch of seed vertices which are highly relevant with the target topic “diabetes”, such as the examination “blood sugar”, the symptom “polydipsia” and etc. These seeds are chosen as the initial central vertices, denoted by $v^{(1)}_{c}$.
- **Expectation step** In iteration *t*, based on the selected central vertices, we expect to find the vertices which are close enough to the centre. Thus, all the neighbour vertices of $v^{(t)}_{c}$ are chosen, denoted by $v^{(t)}_{k}$. Then, we can infer an induced subgraph $G^{(t)}_{k}$ from *G*_*d*_ whose vertex set is $v^{(t)}_{k} \bigcup v^{(t)}_{c}$.
- **Maximization step** Given the induced subgraph $G^{(t)}_{k}$, each vertice *v*_*i*_ is assigned a new eigenvector centrality score *c*(*v*_*i*_). A threshold *ϕ*_*c*_ is employed to decide a new group of the probable central vertices. Those vertices in $G^{(t)}_{k}$ with higher centrality score than *ϕ*_*c*_ are selected, denoted by $v^{(t+1)}_{c}$. Then we start the next iteration.

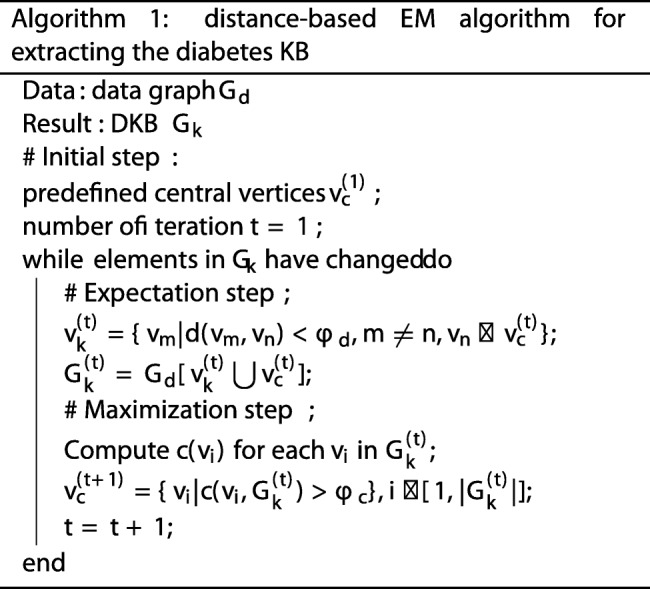


The iterative process continues until EM converges, and finally returns the target DKB *G*_*k*_. Note that both *ϕ*_*c*_ and *ϕ*_*d*_ are set 0.01 in this paper.

## Results

### Data Statistics

We select eight popular vertical portals in China as data sources to construct DKB. Details are illustrated in Table 1. The data was crawled in November, 2017. All the data we collected are Chinese resources. However, our proposed approach is language independent so that it can be applied to other popular resources in the same way. For simplicity, we denote class Disease shortly by “Dis”, Symptom by “Symp”, Western Medicine by “WM”, Traditional Chinese Medicine by “TCM”, Examination by “Exam”, Department by “Dept”, and Body Structure by “Body”.

**Table 1:** Data distribution

	Dis	Symp	WM	TCM	Exam	Dept	Body	Instances	Edges
39Health	*√*	*√*	*√*	*√*	*√*	*√*	*√*	30,720	154,579
99Health	*√*	*√*			*√*	*√*	*√*	3429	24,511
FhHealth	*√*	*√*	*√*	*√*		*√*	*√*	18,776	122,899
Familydoctotr	*√*	*√*	*√*	*√*		*√*	*√*	20,539	11,2106
GlobalHospital	*√*	*√*				*√*	*√*	3624	12,976
PcBaby	*√*	*√*	*√*	*√*		*√*	*√*	5810	13,181
JianKe	*√*	*√*	*√*	*√*	*√*	*√*	*√*	16,866	55,132
120Ask	*√*	*√*	*√*	*√*	*√*	*√*	*√*	45,576	175,528
Overall KB	*√*	*√*	*√*	*√*	*√*	*√*	*√*	76,262	595,515
DKB	*√*	*√*	*√*	*√*	*√*	*√*	*√*	7703	96,041

All the selected vertical portals provided pages describing diseases, symptoms, departments and body stuctures. However, two of them did not provide data about medicine while the knowledge on medical examinations can only be found in six sources. “Quality of the Extracted Knowledge from Vertical Portals” section will discuss the quality of these extracted triples as well as the whole DKB in detail.

We also carry out a detailed statistics of the scale of the KB constructed from each portal. On the right side of the table, we list the number of total instances and edges. Compared with each single data source, we also list the statistics about our overall KB as well as the DKB. From Table 1, we have the following findings:

- The portal contributes most to our overall KB is “120ask”. It contains 45,576 instances and 175,528 edges, only about a half of the instances in the overall KB and a quarter of the edges as well. This proves the advantages of collecting data from different sources.
- The number of instances added from all sources is 145,340, which is far larger than the number of distinct instances 76,262 in our overall KB. This shows the extent of duplications between different data sources.

### Knowledge Fusion Performance

**Fig. 5:**
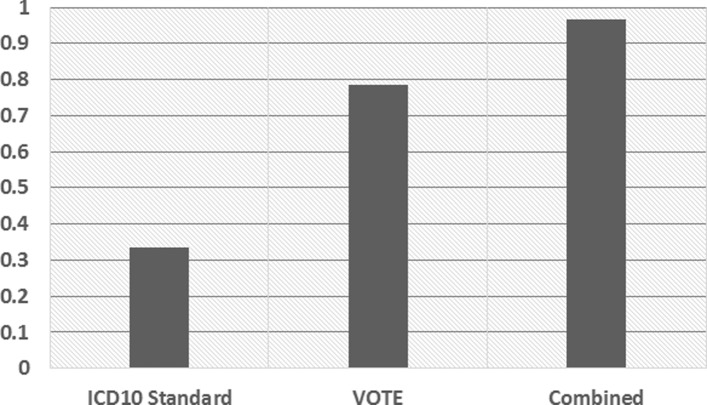
Class cleansing performance

In this section, we evaluate the effectiveness of the knowledge fusion step described in “Knowledge Fusion” section. Particularly, we verify the performance of both class cleansing and instance matching. Seven judges participated in our evaluation and evaluated a total number of 1675 instances with conflicting classes in the overall KB. Each of them was presented with randomly selected instances to assess the accuracy. Figure 5 gives the comparison results.

We apply different fusion strategies to resolve the conflicts. One baseline only considers using the ICD10 standard code to make a distinction between diseases and other classes. It manages to deal with a small portion of the conflicting instances. However, in most cases, the labels of instances can not be exactly matched to the standard names in the ICD10 list. These labels are usually presented in abbreviations which are more commonly used in the online sites. On the other hand, when we adopt the voting strategy, the coverage increases significantly. This indicates that trusting the class with a majority of vertical portals can actually help resolve the conflicts. In addition, the voting strategy can also deal with conflicts except for the disease-symptom conflicts.

The above two baselines can be considered as complementary method so that our proposed algorithm combines the strengths of both of them, which has gained a further performance increase. Bad cases mainly focus on those low frequency instances. Since we only collect knowledge from eight vertical portals, we do not have sufficient evidences to correct each low frequency and conflict instance. However, we can append more trustworthy vertical portals or some third-party knowledge bases to further improve the data quality, which can be tried in our future work.

**Fig. 6:**
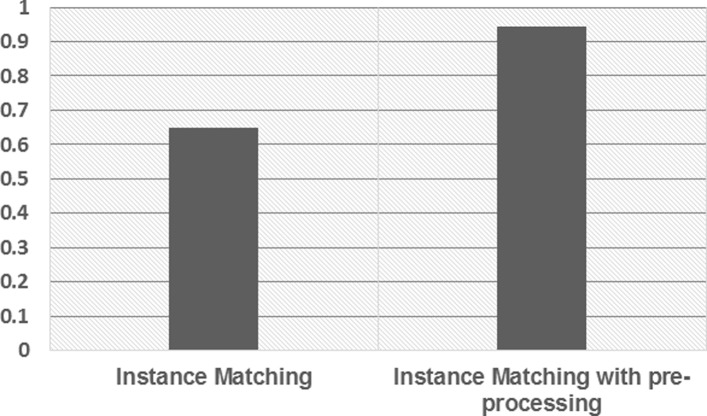
Instance matching performance

Then we evaluate the performance of instance matching. Another 1812 matched pairs from the overall KB were evaluated by the seven judges. Each of them was required to judge whether the given two instances can be aligned. We compare the matching results with an extension which further considers class cleansing as the pre-processing step. Figure 6 shows results of the experiment.

The method achieves a very low accuracy when directly applying a state-of-the-art instance matching algorithm on the extracted data from vertical portals. It is obvious that two instances under different classes can not be matched even though they have the same label. Thus, after we cleanse and correct the conflicting instances, the accuracy increases significantly. This indicates that the class cleansing step can actually help filter the noise data extracted from the Web resources.

### Convergence of the distance-based EM algorithm

**Fig. 7:**
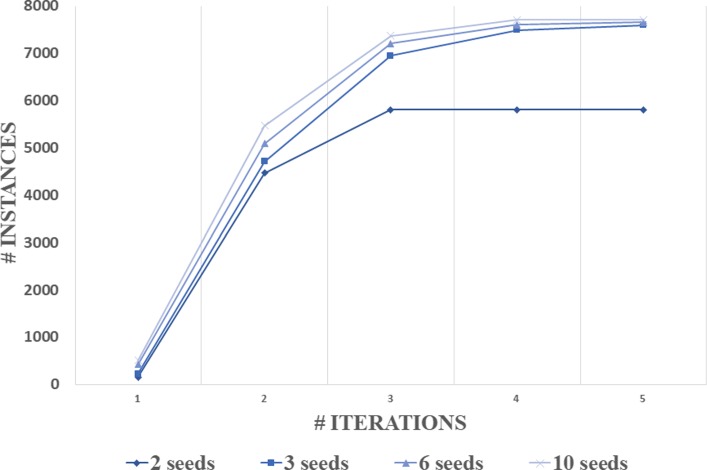
Convergence of the distance-based EM algorithm

In “Extracting DKB from the Overall KB” section, we describe a distance-based EM algorithm with an initial step for extracting a subset from the overall KB. The initial strategy is usually rewarding for the final results in the EM based algorithm. We experimented with a few number of seeds to start the EM iterations and Fig. 7 reports the performance. It turns out that the number of seeds does not make much difference if we choose 2, 3, 6 or 10. The reason is quite clear that after the first and second iterations, most of the core instances in DKB have been already included in the induced subgraph. Moreover, the central vertices are also allowed to be changed. Thus, if a few diabetes-related instances happen to be not included in the initial sets, EM will slowly correct the situation, i.e., moving them to the central of the induced subgraph due to their strong connections with all the other included instances. We also find that running more than four iterations of the proposed EM algorithm dose not significantly increase the scale of DKB. Thus, we use four iterations of EM after the initialization to save computation. We receive totally 7703 instances with 96,041 edges in our final DKB.

### Quality of the Extracted Knowledge from Vertical Portals

In order to evaluate the quality of the extracted knowledge, we use the accuracy of triples for correctness evaluation. Each sampled triple was evaluated by seven judges, and the evaluators judged the correctness of triples according to their own knowledge. We sampled 417 triples from 595,515 triples in the overall KB and the accuracy is 98.1%. We also sampled 423 triples from 9232 triples in the DKB and the accuracy is 95.91%.

Bad cases mainly rely on a small portion of the self-conflicted descriptions in some of the portals. For example, the instance “ (Hydronephrosis)” is classified as a symptom in the main index of “ (PcBaby)”. However, it is also described as a related disease of itself on its own page, which may lead to another triple declaring “ (Hydronephrosis)” as a disease. That is to say, each portal may describe some conflicting classes by themselves. Since each instance is assigned an unique identifier in its URI, it should only belong to one class. Thus, some correct data may be replaced with wrong ones according to the self-conflicted knowledge descriptions.

## Discussions

Our approach was proposed for constructing a DKB via mining the Web, which included a knowledge extraction method, a knowledge fusion method and subset extraction method. Compared with previous work, the new approach is focus on Chinese diabetes knowledge and builds a unified KB in order to make use of all healthcare information. The result of data statistics shows the advantages of collecting data from different sources, and the extent of duplications between different data sources.

To demonstrate the effectiveness of knowledge fusion, we verify the performance of both class cleansing and instance matching, and the result of improved approach shows a significantly increases. In order to evaluate the quality of the extracted knowledge, we use the accuracy of triples for correctness evaluation, the accuracy of overall KB is 98.1%, and 95.91% to the DKB. To understand the weakness of our approach for further improvement, we have analyzed the result and found that bad cases mainly rely on a small portion of the self-conflicted descriptions in some of the portals.

## Conclusions

In this paper, we introduced an approach to constructing DKB. A knowledge extraction and fusion pipeline was first used to extract semi-structured data from vertical portals and further fuse individual KBs into a unified knowledge base. After that, we developed a distance based Expectation-Maximization algorithm to extract a subset from the overall knowledge base forming the target DKB. Experiments showed that the data in DKB are rich and of high-quality. In the future, we consider to integrate more data sources or third-party data sets to enrich our knowledge base. The predefined relations can also be rewritten into finer-grained ones in order to capture more details.

## Abbreviations

Body: Body structure
Dept: Department
Dis: Disease
DKB: Diabetes-centric knowledge base
Exam: Examination
EM: Expection-maximization
EMRs: Electronic medical records
KB: Knowledge base
Symp: Symptom
TCM: Traditional chinese medicine
WM: Western medicine

## References

[1] Cebul RD, Love TE, Jain AK, Hebert CJ (2011). Electronic health records and quality of diabetes care. N Engl J Med.

[2] Yin S, Chen D, Le J. Deep neural network based on translation model for diabetes knowledge graph. In: 2017 Fifth International Conference on Advanced Cloud and Big Data (CBD). IEEE: 2017. p. 318–323. 10.1109/cbd.2017.62.

[3] Yang Z, Yang J, Liu W, Wu L, Xing L, Wang Y, Fan X, Cheng Y. T2d@ZJU: a knowledgebase integrating heterogeneous connections associated with type 2 diabetes mellitus. Database. 2013;2013. 10.1093/database/bat052. 10.1093/database/bat052 PMC3708620 23846596

[4] Gopinath K, Jayakumararaj R, Karthikeyan M (2015). DAPD: A knowledgebase for diabetes associated proteins. IEEE/ACM Trans Comput Biol Bioinforma.

[5] Rotmensch M, Halpern Y, Tlimat A, Horng S, Sontag D. Learning a health knowledge graph from electronic medical records. Sci Reports. 2017; 7(1). 10.1038/s41598-017-05778-z. 10.1038/s41598-017-05778-z PMC5519723 28729710

[6] Resource Description Framework (RDF). https://www.w3.org/2001/sw/wiki/RDF. Accessed 1 Feb 2019.

[7] RDF Schema 1.1. http://www.w3.org/TR/rdf-schema/. Accessed 1 Feb 2019.

[8] Web Ontology Language (OWL). https://www.w3.org/OWL/. Accessed 1 Feb 2019.

[9] Frequently Asked Questions About Diabetes. http://www.webmd.com/diabetes/diabetes-faq. Accessed 1 Feb 2019.

[10] Schmitt A, Gahr A, Hermanns N, Kulzer B, Huber J, Haak T (2013). The diabetes self-management questionnaire (DSMQ): development and evaluation of an instrument to assess diabetes self-care activities associated with glycaemic control. Health Qual Life Outcome.

[11] Abadi DJ, Marcus A, Madden S, Hollenbach KJ. Scalable semantic web data management using vertical partitioning In: Koch C, Gehrke J, Garofalakis MN, Srivastava D, Aberer K, Deshpande A, Florescu D, Chan CY, Ganti V, Kanne C, Klas W, Neuhold EJ, editors. Proceedings of the 33rd International Conference on Very Large Data Bases, University of Vienna, Austria, September 23-27, 2007. ACM: 2007. p. 411–422. http://www.vldb.org/conf/2007/papers/research/p411-abadi.pdf.

[12] Daskalaki E, Flouris G, Fundulaki I, Saveta T (2016). Instance matching benchmarks in the era of linked data. J Web Semant.

[13] Mausam M. Open information extraction systems and downstream applications. In: Proceedings of the Twenty-Fifth International Joint Conference on Artificial Intelligence, IJCAI’16. AAAI Press: 2016. p. 4074–4077. http://dl.acm.org/citation.cfm?id=3061053.3061220. Accessed 1 Feb 2019.

[14] Jens L, Robert I, Max J, Anja J, Dimitris K, Mendes PN, Sebastian H, Mohamed M, van Kleef P, Auer S (2015). Dbpedia–a large-scale, multilingual knowledge base extracted from wikipedia. Semant Web.

[15] Rebele T, Suchanek F, Hoffart J, Biega J, Kuzey E, Weikum G (2016). YAGO: A multilingual knowledge base from wikipedia, wordnet, and geonames. Lecture Notes in Computer Science.

[16] Niu X, Sun X, Wang H, Rong S, Qi G, Yu Y (2011). Zhishi.me - weaving chinese linking open data. The Semantic Web – ISWC 2011.

[17] Hu F, Shao Z, Ruan T (2014). Self-supervised chinese ontology learning from online encyclopedias. Sci World J.

[18] Sogou. https://www.sogou.com/. Accessed 1 Feb 2019.

[19] Baidu. http://www.baidu.com. Accessed 1 Feb 2019.

[20] Bodenreider O (2004). The unified medical language system (UMLS): integrating biomedical terminology. Nucleic Acids Res.

[21] Stearns M, Price C, Spackman K, Wang A (2001). Snomed clinical terms: overview of the development process and project status. Proc/AMIA Annu Symp AMIA Symp.

[22] Law V, Knox C, Djoumbou Y, Jewison T, Guo AC, Liu Y, Maciejewski A, Arndt D, Wilson M, Neveu V, Tang A, Gabriel G, Ly C, Adamjee S, Dame ZT, Han B, Zhou Y, Wishart DS (2013). DrugBank 4.0: shedding new light on drug metabolism. Nucleic Acids Res.

[23] Kuhn M, Letunic I, Jensen LJ, Bork P (2015). The SIDER database of drugs and side effects. Nucleic Acids Res.

[24] Ernst P, Siu A, Weikum G. KnowLife: a versatile approach for constructing a large knowledge graph for biomedical sciences. BMC Bioinformatics. 2015; 16(1). 10.1186/s12859-015-0549-5. 10.1186/s12859-015-0549-5 PMC4448285 25971816

[25] Smink LJ (2004). T1dbase, a community web-based resource for type 1 diabetes research. Nucleic Acids Res.

[26] Agrawal S, Dimitrova N, Nathan P, Udayakumar K, Lakshmi SS, Sriram S, Manjusha N, Sengupta U (2008). T2d-db: An integrated platform to study the molecular basis of type 2 diabetes. BMC Genomics.

[27] Lim JE, Hong K-W, Jin H-S, Kim YS, Park HK, Oh B. Type 2 diabetes genetic association database manually curated for the study design and odds ratio. BMC Med Inform Decis Making. 2010;10(1). 10.1186/1472-6947-10-76. 10.1186/1472-6947-10-76 PMC3022779 21190593

[28] Yang Z, Yang J, Liu W, Wu L, Xing L, Wang Y, Fan X, Cheng Y. T2d@ZJU: a knowledgebase integrating heterogeneous connections associated with type 2 diabetes mellitus. Database. 2013;2013. 10.1093/database/bat052. 10.1093/database/bat052 PMC3708620 23846596

[29] 39Health. http://www.39.net/. Accessed 1 Feb 2019.

[30] 99Health. http://www.99.com.cn/. Accessed 1 Feb 2019.

[31] FhHealth. http://www.fh21.com.cn/. Accessed 1 Feb 2019.

[32] Familydoctotr. http://www.familydoctor.com.cn/. Accessed 1 Feb 2019.

[33] GlobalHospital. http://www.qqyy.com/. Accessed 1 Feb 2019.

[34] PcBaby. http://www.pcbaby.com.cn/. Accessed 1 Feb 2019.

[35] JianKe. http://www.jianke.com/. Accessed 1 Feb 2019.

[36] 120Ask. http://www.120ask.com/. Accessed 1 Feb 2019.

[37] Dalvi N, Kumar R, Soliman M (2011). Automatic wrappers for large scale web extraction. Proc VLDB Endowment.

[38] Ciravegna F, Gentile AL, Zhang Z (2012). LODIE: linked open data for web-scale information extraction. SWAIE.

[39] D, 2RQ Accessing Relational Databases as Virtual RDF Graphs. http://d2rq.org/.

[40] JeÅek P, MouÄek R. Semantic framework for mapping object-oriented model to semantic web languages. Front Neuroinformatics. 2015; 9. 10.3389/fninf.2015.00003. 10.3389/fninf.2015.00003 PMC4340193 25762923

[41] Eisenberg V, Kanza Y. D2rq/update: Updating relational data via virtual rdf. In: Proceedings of the 21st International Conference Companion on World Wide Web. ACM Press: 2012. 10.1145/2187980.2188095.

[42] The 10th Revision of the International Statistical Classification of Diseases and Related Health Problems. http://www.who.int/classifications/apps/icd/icd10online/. Accessed 1 Feb 2019.

[43] Chinese Descriptions and Aliases of ICD-10 in Wikipedia. https://zh.wikipedia.org/wiki/ICD-10. Accessed 1 Feb 2019.

[44] Euzenat J, Shvaiko P. Ontology Matching: Springer Berlin Heidelberg; 2013. 10.1007/978-3-642-38721-0.

[45] Dempster AP, Laird NM, Rubin DB (1977). Maximum likelihood from incomplete data via the em algorithm. J R Stat Soc Ser B Methodol.

